# A new species of the genus *Anteon* Jurine (Hymenoptera, Dryinidae) from Thailand

**DOI:** 10.3897/zookeys.504.9333

**Published:** 2015-05-19

**Authors:** Massimo Olmi, Zaifu Xu, Adalgisa Guglielmino

**Affiliations:** 1 Tropical Entomology Research Center, Viterbo, Italy; 2 Department of Entomology, South China Agricultural University, Guangzhou, Guangdong, P.R. China; 3 Department of Agriculture, Forests, Nature and Energy, University of Tuscia, Viterbo, Italy

**Keywords:** Taxonomy, *Anteon huettingeri*, Oriental region, key, Nan Province, Anteoninae

## Abstract

A new species of *Anteon* Jurine, 1807 is described from Thailand, Nan Province: *Anteon huettingeri* **sp. n.** Morphologically the new species is similar to *Anteon borneanum* Olmi, 1984, *Anteon jurineanum* Latreille, 1809, *Anteon insertum* Olmi, 1991, *Anteon yasumatsui* Olmi, 1984, *Anteon sarawaki* Olmi, 1984, *Anteon thai* Olmi, 1984 and *Anteon krombeini* Olmi, 1984, but it is clearly different for the numerous sensorial processes present on the inner side of the paramere; these processes are absent in the other above species. Published identification keys to the Oriental species of *Anteon* are modified to include the new species.

## Introduction

Dryinidae (Hymenoptera, Chrysidoidea) are parasitoids of leafhoppers, planthoppers and treehoppers (Hemiptera, Auchenorrhyncha) (Guglielmino and Bückle 2003, 2010; Guglielmino et al. 2006, 2013; Guglielmino and Olmi 2013; Guglielmino and Virla 1998). *Anteon* Jurine, 1807 is a genus that is present in all zoogeographical regions (Olmi 1984; Xu et al. 2013; Olmi and Virla 2014). In total 423 species have been described from all continents (Olmi and Virla 2014) and the genus was revised at world level by Olmi (1984, 1991) and in the Oriental and Neotropical regions by Xu et al. (2013) and Olmi and Virla (2014) respectively.

The species of *Anteon* inhabiting the Oriental region have been recently studied by Xu et al. (2013). In total 149 species have been described from the Oriental region (Guglielmino and Olmi 2013; Xu et al. 2013).

*Anteon* species are parasitoids of leafhoppers belonging to Cicadellidae (Guglielmino et al. 2013). As in almost all dryinids, females of *Anteon* have a chelate protarsus. Chelae are used to capture and restrain the host during oviposition and host-feeding (Olmi 1984, 1994).

In 2014 we examined additional specimens of *Anteon* from Thailand and discovered a new species described in this paper.

## Material and methods

The descriptions follow the terminology used by Olmi (1984) and Xu et al. (2013). The measurements reported are relative, except for the total length (head to abdominal tip, without antennae), which is expressed in millimetres. The following abbreviations are used in the descriptions: POL is the distance between the inner edges of the two lateral ocelli; OL is the distance between the inner edges of a lateral ocellus and the median ocellus; OOL is the distance from the outer edge of a lateral ocellus to the compound eye; OPL is the distance from the posterior edge of a lateral ocellus to the occipital carina; TL is the distance from the posterior edge of an eye to the occipital carina.

The types of all Oriental species of *Anteon* have been previously examined by the authors.

The type specimen described in this paper is deposited in the collection of the Oberösterreichisches Landesmuseum, Linz, Austria (OLL).

The description of the new species is based on the study of only a single specimen. The authors are aware that descriptions of new taxa should normally be based on more individuals. However, Dryinidae are so rare that it is uncommon to collect more than one specimen of each species. In addition, on the basis of the experience and knowledge of the authors, the new species is sufficiently delimited by unique characters to justify its description.

## Results

### Genus Anteon

Taxon classificationAnimaliaHymenopteraDryinidae

Jurine, 1807

Anteon Jurine, 1807: 302. Type species: *Anteon jurineanum* Latreille, 1809, by subsequent monotypy.

#### Diagnosis.

Female: Fully winged; rarely brachypterous; occipital carina complete; palpal formula 6/3; antenna without rhinaria; forewing with three cells enclosed by pigmented veins (costal, median and submedian); forewing with stigmal vein and pterostigma; distal part of stigmal vein much shorter than proximal part, occasionally slightly shorter, as long as, or longer than proximal part; propodeum usually with transverse keel between dorsal and posterior surface; protarsus chelate; inner side of enlarged claw with proximal prominence bearing one long bristle; tibial spurs 1/1/2. Male: Fully winged; rarely brachypterous; occipital carina complete; vertex of head usually without two oblique keels connecting posterior ocelli to occipital carina; palpal formula 6/3; forewing with three cells enclosed by pigmented veins (costal, median and submedian); forewing with stigmal vein and pterostigma; distal part of stigmal vein much shorter than proximal part, occasionally slightly shorter, as long as, or longer than proximal part; pterostigma less than four times as long as broad; propodeum usually with transverse keel between dorsal and posterior surface; paramere without inner branch wrapping penis; tibial spurs 1/1/2.

### Anteon huettingeri

Taxon classificationAnimaliaHymenopteraDryinidae

Olmi, Xu & Guglielmino sp. n.

http://zoobank.org/9DAA0C1A-15FE-40C1-9C9F-19C9A6676F8F

#### Diagnosis.

**Figure 1.**
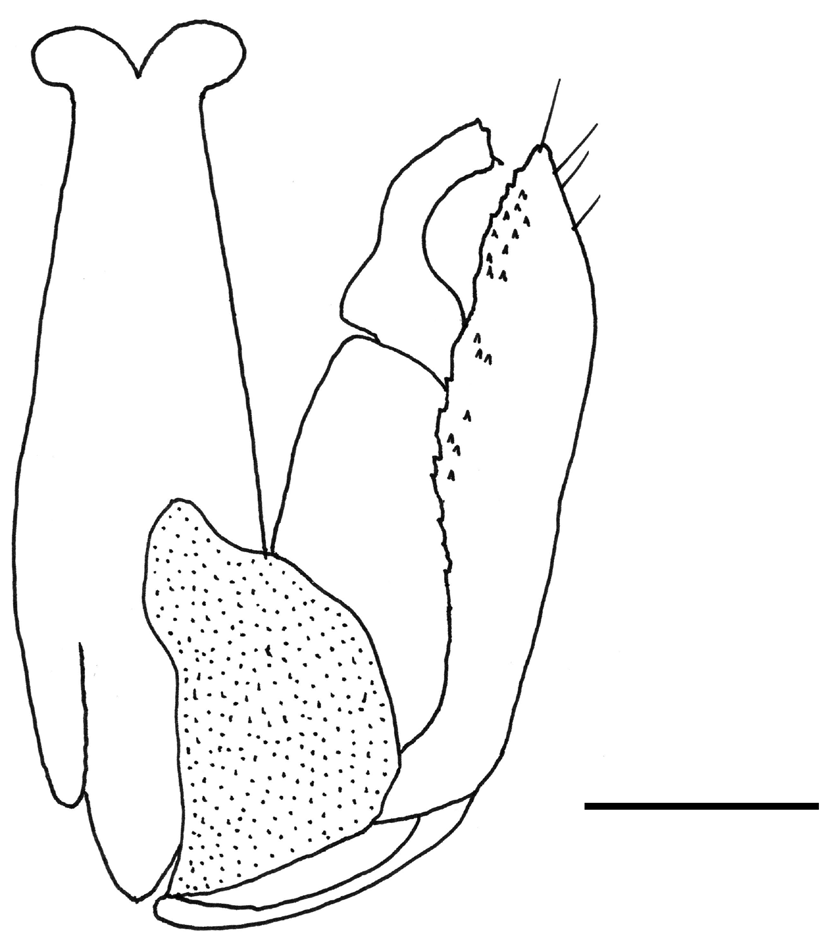
*Anteon huettingeri* Olmi, Xu & Guglielmino, sp. n.: male genitalia (left half removed). Scale bar = 0.06 mm.

Male with antenna filiform; scutum very slightly granulated and finely punctate; posterior surface of propodeum without longitudinal keels; paramere without distal inner process, with inner side provided with many sensorial processes (Fig. 1).

#### Description.

**Figure 2.**
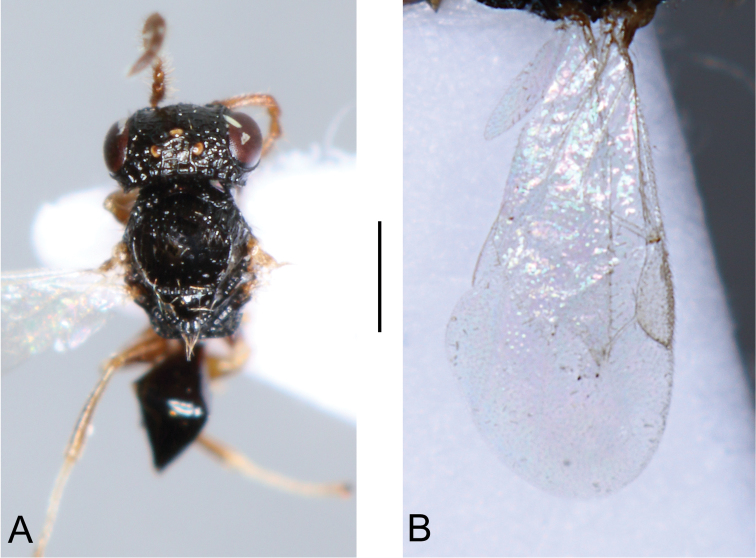
*Anteon huettingeri* Olmi, Xu & Guglielmino, sp. n.: **A** dorsal side **B** forewing. Scale bar = 0.67 mm (**A**), 0.41 mm (**B**).

**Male.** Fully winged (Fig. 2A). Length 2.2 mm. Head black, except mandible testaceous. Antenna testaceous. Mesosoma black. Metasoma brown. Legs testaceous, except metacoxa partly black. Antenna filiform. Antennal segments in following proportions: 11:6:6:5:5:6:5:5:5:7. Head dull, granulated and reticulate rugose. Face with two lateral keels around orbits directed towards antennal toruli. Vertex with two short oblique keels from posterior ocelli to occipital carina. Occipital carina complete. Frontal line complete. Vertex with POL = 6; OL = 3; OOL = 4; OPL = 3; TL = 4; greatest breadth of posterior ocelli as long as OPL. Scutum shiny, very slightly granulated, finely punctate, unsculptured among punctures. Notauli incomplete, reaching approximately 0.4 × length of scutum. Scutellum and metanotum unsculptured, shiny. Propodeum with strong transverse keel between dorsal and posterior surface. Dorsal surface of propodeum reticulate rugose. Posterior surface of propodeum without longitudinal keels, with median area granulated and lateral areas reticulate rugose. Forewing (Fig. 2B) hyaline, without dark transverse bands. Distal part of stigmal vein much shorter than proximal part (2:5). Paramere (Fig. 1) without distal inner process, with inner side provided with many small sensorial processes. Tibial spurs 1/1/2.

**Female.** Unknown.

#### Material examined.

**Holotype:** male, Thailand, Nan Province, outside Mae Charim National Park gate, 18°36.00'N, 100°58.34'E, 260 m, 13.v.2012, E. & J. Hüttinger leg. (OLL).

#### Distribution.

Thailand.

#### Hosts.

Unknown.

#### Etymology.

The species is named after the collector, Dr Ernst Hüttinger.

#### Remarks.

Because of the antenna filiform, the scutum neither rugose nor sculptured by irregular keels, the posterior surface of the propodeum without longitudinal keels, the paramere without distal inner process, the new species is similar to *Anteon borneanum* Olmi, 1984, *Anteon insertum* Olmi, 1991, *Anteon jurineanum* Latreille, 1809, *Anteon krombeini* Olmi, 1984, *Anteon sarawaki* Olmi, 1984, *Anteon thai* Olmi, 1984 and *Anteon yasumatsui* Olmi, 1984. The main difference between *Anteon huettingeri* and all other species is centered on the structure of the inner side of the paramere (with many sensorial processes in *Anteon huettingeri* (Fig. 1); without sensorial processes in the other species (Plates 20 C, 30 D, 31 C, 31 D, 32 C, 43 B, 48 A, 50 E in Xu et al. 2013)). In the key to the males of Oriental *Anteon* published by Xu et al. (2013), the new species can be included by replacing couplet 9 as follows:

**Table:** 

9	Inner side of paramere with many small sensorial processes (Fig. 1)	***Anteon huettingeri* Olmi, Xu & Guglielmino, sp. n.**
–	Inner side of paramere without sensorial processes (Plates 20 C, 30 D, 31 C, 31 D, 32 C, 43 B, 48 A, 50 E in Xu et al. 2013)	**9**’
9’	Scutum granulated	**10**
–	Scutum punctate, or unsculptured, not granulated; occasionally scutum partly reticulate rugose	**11**
10	Paramere much shorter than penis (Plate 20C in Xu et al. 2013)	***Anteon borneanum* Olmi**
–	Paramere about as long as penis (Plate 31C, D in Xu et al. 2013)	***Anteon jurineanum* Latreille**
11	Head punctate, unsculptured among punctures	***Anteon insertum* Olmi**
–	Head granulated, or rugose, or with irregular keels	**12**
12	Head dull, smooth, granulated	***Anteon yasumatsui* Olmi**
–	Head shiny, rugose, with areolae and irregular keels	**13**
13	Notauli almost reaching posterior margin of scutum	***Anteon sarawaki* Olmi**
–	Notauli reaching at most 0.5 length of scutum	**14**

## Conclusion

Xu et al. (2013) recorded 71 species of Dryinidae from Thailand. They belong to the following genera: *Aphelopus* Dalman, 1823 (seven species), *Crovettia* Olmi, 1984 (one species), *Anteon* Jurine, 1807 (26 species), *Deinodryinus* Perkins, 1907 (two species), *Bocchus* Ashmead, 1893 (three species), *Thaumatodryinus* Perkins, 1905 (two species), *Dryinus* Latreille, 1804 (13 species), *Pseudodryinus* Olmi, 1991 (one species), *Neodryinus* Perkins, 1905 (five species), *Echthrodelphax* Perkins, 1903 (three species), *Haplogonatopus* Perkins, 1905 (one species) and *Gonatopus* Ljungh, 1810 (seven species). With the description of the above new species the number of species now known in Thailand is 72.

In comparison with the 193 species recorded in China by He and Xu (2002) and the 62 and 40 listed respectively in India and Laos (Xu et al. 2013), the dryinid fauna of Thailand is poorly known. Some genera such as *Gonatopus* (with only seven species listed) are clearly under studied.

## Supplementary Material

XML Treatment for Anteon

XML Treatment for Anteon huettingeri

## References

[B1] Ashmead WH (1893) Monograph of the North American Proctotrypidae. Bulletin of the United States National Museum 45: 1–472. doi: 10.5479/si.03629236.45.1

[B2] Dalman CR (1823) Analecta entomologica. Typis Lindhianis, Holmiae, Sweden, 104 pp. doi: 10.5962/bhl.title.66069

[B3] Guglielmino A Bückle C (2003) Description of larval instars of *Neodryinus typhlocybae* (Ashmead, 1893) (Hymenoptera Dryinidae), with remarks on its biology. Mitteilungen aus dem Museum fuer Naturkunde in Berlin - Deutsche Entomologische Zeitschrift 50(1): 143–150. doi: 10.1002/mmnd.20030500114

[B4] Guglielmino A Bückle C (2010) Description of larval instars of *Mystrophorus formicaeformis* Ruthe (Hymenoptera: Dryinidae). Zootaxa 2602: 57–66.

[B5] Guglielmino A Bückle C Moya-Raygoza G (2006) Description of the larval instars of *Gonatopus bartletti* Olmi, 1984 (Hymenoptera: Dryinidae). Zootaxa 1226: 51–60.

[B6] Guglielmino A Olmi M (2013) Description of *Anteon seramense* (Hymenoptera: Dryinidae), a new species from Indonesia. Florida Entomologist 96(2): 598–601. doi: 10.1653/024.096.0226

[B7] Guglielmino A Olmi M Bückle C (2013) An updated host-parasite catalogue of world Dryinidae (Hymenoptera: Chrysidoidea). Zootaxa 3740: 1–113. doi: 10.11646/zootaxa.3740.1.1 25112881 10.11646/zootaxa.3740.1.1

[B8] Guglielmino A Virla EG (1998) Postembryonic development of *Gonatopus lunatus* Klug (Hymenoptera: Dryinidae: Gonatopodinae), with remarks on its biology. Annales de la Société entomologique de France (N. S.) 34(3): 321–333.

[B9] He J Xu Z (2002) HymenopteraDryinidae (Fauna Sinica 29). Science Press, Beijing, China, 464 pp.

[B10] Jurine L (1807) Nouvelle méthode de classer les Hyménoptères et les Diptères, 1. Hyménoptères. Paschoud, Genève, Switzerland, 319 pp.

[B11] Latreille PA (1804) Nouvelle dictionnaire d’Histoire naturelle, 24 F. Dufart, Paris, France, 104 pp.

[B12] Latreille PA (1809) Genera Crustaceorum et Insectorum secundum ordinem naturalem in familias disposita, 4 Amand Koenig, Parisiis et Argentorati, 399 pp.

[B13] Ljungh SJ (1810) *Gonatopus*, novum insectorum genus. Beiträge zur Naturkunde 2: 161–163.

[B14] Olmi M (1984) A revision of the Dryinidae (Hymenoptera). Memoirs of the American Entomological Institute 37: 1–1913.

[B15] Olmi M (1991) Supplement to the revision of the world Dryinidae (Hymenoptera Chrysidoidea). Frustula entomologica (1989) (N. S.) 12(25): 109–395.

[B16] Olmi M (1994) The Dryinidae and Embolemidae (Hymenoptera: Chrysidoidea) of Fennoscandia and Denmark (Fauna Entomologica Scandinavica 30). E. J. Brill, Leiden, Netherlands, 100 pp.

[B17] Olmi M Virla EG (2014) Dryinidae of the Neotropical Region (Hymenoptera: Chrysidoidea). Zootaxa 3792(1): 1–534. doi: 10.11646/zootaxa.3792.2.1 24869997 10.11646/zootaxa.3792.2.1

[B18] Perkins RCL (1903) The leafhopper of the sugar cane. Territory of Hawaii, Board of Agriculture and Forest, Division of Entomology, Bulletin 1: 1–38.

[B19] Perkins RCL (1905) Leafhoppers and their natural enemies (Pt. i. Dryinidae). Report of Work of the Experiment Station of the Hawaiian Sugar Planters’ Association, Division of Entomology, Bulletin 1(I): 1–69.

[B20] Perkins RCL (1907) Parasites of leaf-hoppers. Report of Work of the Experiment Station of the Hawaiian Sugar Planters’ Association, Division of Entomology, Bulletin 4: 5–59.

[B21] Xu Z Olmi M He J (2013) Dryinidae of the Oriental region (Hymenoptera: Chrysidoidea). Zootaxa 3614: 1–460. doi: 10.11646/zootaxa.3900.1.1 24759692 10.11646/zootaxa.3614.1.1

